# Gene conversion and purifying selection of a placenta-specific ERV-V envelope gene during simian evolution

**DOI:** 10.1186/1471-2148-8-266

**Published:** 2008-09-30

**Authors:** Anders L Kjeldbjerg, Palle Villesen, Lars Aagaard, Finn Skou Pedersen

**Affiliations:** 1 Department of Molecular Biology, University of Aarhus, DK-8000 Aarhus C, Denmark; 2 Bioinformatics Research Center, University of Aarhus, DK-8000 Aarhus C, Denmark

## Abstract

**Background:**

Most human endogenous retroviruses (HERVs) invaded our genome at least 25 million years ago. The majority of the viral genes are degenerated, since no selection preserves them within the genome. However, a few intact and very old HERV genes exist, and likely are beneficial for the host. We here address evolutionary aspects of two HERV-V envelope genes, *ENVV1 *and *ENVV2*, located in tandem and containing a long open reading frame.

**Results:**

The *ENVV2 *gene is preserved with an intact reading frame during simian evolution, but none of the *ENVV *genes are found in the prosimian species tested. While we observe many transposon insertions in the *gag *and *pol *regions of the ERV-V2 provirus, the *ENVV2 *genes have escaped transposon crossfire in all species tested. Additional analysis of nucleotide substitutions provides further strong evidence of purifying selection on the *ENVV2 gene *during primate evolution. The other copy, *ENVV1*, seems to be involved in gene conversion of the major part of the envelope. Furthermore, *ENVV1 *and *ENVV2 *show placenta-specific expression in human and a baboon species.

**Conclusion:**

Our analyses show that ERV-V entered our genome after the split between simian and prosimian primates. Subsequent purifying selection and gene conversion have preserved two copies of the *ENVV *envelope gene in most species. This is the first case of gene conversion involving long open reading frames in HERVs. Together with the placenta-specific expression of the human and baboon *ENVV1 *and *ENVV2 *envelope genes, these data provide strong evidence of a beneficial role for the host.

## Background

Upon retrovirus infection of somatic cells, the integrated provirus will not be passed on to the host progeny as a part of the genome. However, following infection of the germ line, the integrated provirus will be transmitted to the offspring. Consequently, progeny developed from infected germ cells will carry the provirus as part of their genome, and it will be transmitted vertically through generations as an endogenous retrovirus (ERV). Each independent germ line infection event defines a novel ERV family, which may increase its copy number due to intracellular retrotransposition [1] or extracellularly via re-infection [2], and in the end each ERV infection results in a few to several hundred genomic copies [3]. Most HERVs invaded our genome at least 25 million years ago (mya) [4,5], after separation of Old World and New World monkeys around 43 mya [6]. ERVs that entered the human genome before the split of human (*Homo sapiens*) and chimpanzee *(Pan troglodytes) *are characterized as ancient HERVs. However, some HERVs, characterized as modern HERVs, are human-specific and have entered the human genome after the *Homo sapiens/Pan troglodytes *split. Further, some HERV loci have been reported to show insertional polymorphism in the human genome, even one HERV locus has entered the human genome less than 1 mya [7].

Generally HERV-encoded genes are thought to be inactivated by negative selection, followed by degeneration due to mutational decay during evolution. However, a few HERV loci do still maintain intact open reading frames of viral genes, indicating either recent integration or ongoing purifying selection. No replication-competent HERVs have yet been described, although fully intact members of the HERV-K group have been reported [7]. Nevertheless, trans-complementation and recombination of human HERV-K loci can generate functional HERV-K elements, indicating that human cells still have the potential to produce infectious retrovirus particles [8,9]. However, other mammalian species such as mouse, cat and pig harbor many modern ERVs which are still replication-competent [4].

Intact envelope genes have been shown to be transcribed in several healthy tissues [10], and the conservation in an otherwise degenerated HERV locus has led to speculations about a likely beneficial role for the host. These include (i) protecting the fetus due to immunomodulatory properties via an immunosuppressive domain located in the TM subunit of the envelope [11,12], (ii) preventing present-day retroviral infections by inhibiting cell entry of related exogenous retroviruses that use a common surface receptor, a process called receptor interference in which the receptor-binding-domain of SU blocks the receptor [13,14], or (iii) being used as triggers to provide cell-cell fusion in which the fusion machinery of TM is activated by binding of SU to a cellular receptor. In particular three HERV envelope genes can induce cell-cell fusion *in vitro*, syncytin 1 [15,16], syncytin 2 [17], and EnvPb1 [18]. All three are candidates for having a beneficial function because they are evolutionarily conserved and have undergone purifying selection during primate evolution [17,19,20]. Furthermore all single nucleotide polymorphisms (SNPs) within the three envelope genes are either synonymous or they do not influence fusiogenicity [20,21].

Syncytin 1 and syncytin 2 show placenta-specific expression [10,15,22,23], which may be an implication of a physiological role of HERV envelope proteins in mediating cell-cell fusion in placenta forming the syncytiotrophoblast. In fact inhibition of syncytin 1 in human cytotrophoblasts leads to a decrease in cell fusion [24], indicating a plausible physiological role of syncytin 1 in placenta development. Syncytin 2 might protect the fetus against the mother's immune system due to immunosuppressive properties [11], but so far none of them have been well enough characterized to draw functional conclusions.

During a screen of the human genome for retroviral open reading frames [25], we identified a new group, dubbed HERV-V [18], containing two almost identical envelope genes. HERV-V was recently proposed to be a degenerate syncytin [11]. However, we here demonstrate that selection has preserved at least one of the envelope genes through simian evolution, and that the other envelope gene has been partly preserved by gene conversion.

## Results

**Figure 1:**
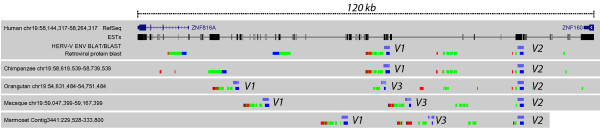
***ENVV2 *in primate genomes**. The location of ERV-V loci on chromosome 19 in five primate species. The *ENVV *genes are shown in light blue whereas similarity to other HERV proteins are coded as gag (red), pol (green) and env (blue).

The two, almost identical, HERV-V envelope genes are both located on chromosome 19q13.41 with a distance between the two loci of ~34 kb (Figure 1) [25]. In order to distinguish the two envelopes from each other, we propose naming one locus HERV-V1 and its envelope *ENVV1 *(chr19: 58209156–58210586, hg18) and the other locus HERV-V2 and its envelope *ENVV2 *(chr19: 58244317–58245921, hg18). *ENVV1 *has a 477 amino-acid long open reading frame and *ENVV2 *has an open reading frame containing 535 amino acids. Variation is only observed in the C-terminus of the genes by a ~60 amino-acid truncation of *ENVV1*, due to a one nucleotide insertion leading to a frame shift. A tBLASTn search shows no other closely related sequences in the human genome and a sensitive BLASTn search using the cross-species LTR consensus show no sign of solitary LTRs. Further, both envelopes show high similarity in the C-terminal part to two other HERV envelope genes: *ERVWE1 *(syncytin 1) and HERV-FRD (syncytin 2) (*Env-FRD*) [25]. Phylogenetic comparison indicates that HERV-V is a small new family of ERVs, most closely related to that of MER66, MER84 and Z69907 families [18].

### The *ENVV *envelope genes are conserved in simians

Using tBLASTn and BLAT we found *ENVV1 *and *ENVV2 *at homologous positions on chromosome 19 in chimpanzee (*Pan troglodytes*), Sumatran orangutan (*Pongo pygmaeus abelii*), rhesus macaque (*Macaca mulatta*) and marmoset (*Callithrix jacchus, Contig3441*) (Figure 1). In all species, the *ENVV2 *gene is intact, whereas the *ENVV1 *gene is preserved only in chimpanzee and rhesus macaque, where it exhibits a full-length ORF with no C-terminal truncation or stop codons. In orangutan, rhesus macaque and marmoset, *ENVV1 *and *ENVV2 *are separated by 45–65 kb and exhibit a third ERV-V locus in between, named ERV-V3. No *ENVV *homologues were detected in any genome more distant than those of New World monkeys.

**Figure 2:**
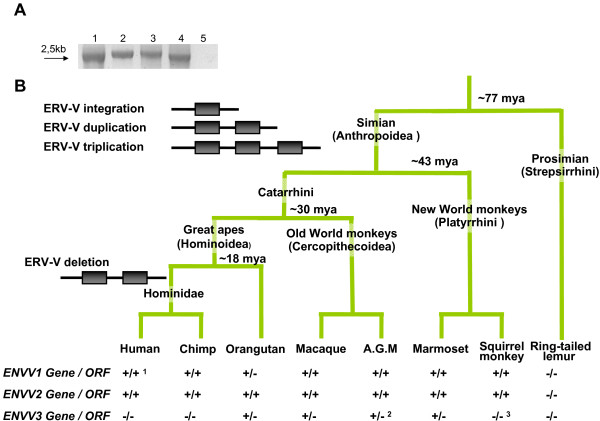
**PCR identification of *ENVV***. A) *ENVV2 *PCR. Lane 1: Human. Lane 2: B. orangutan. Lane 3: Africa green monkey. Lane 4: Squirrel monkey. Lane 5: Ring-tailed lemur. B) Primate phylogeny, with hypothetical integrations indicated. Below each species is indicated whether a given gene/ORF was detected (+) or not (-). Notes: 1) Contains a 60 amino acid C-terminal truncation. 2) Detected by an internal *ENVV *PCR (see methods). 3) Not PCR detected.

Additionally, we screened a primate DNA panel with PCR primers flanking either the *ENVV1 *or *ENVV2 *envelope genes (Figure 2A) and were able to amplify the expected 2.5-kb amplicon from *Hominoidea*, Old World monkeys and New World monkeys (Borneo orangutan (*Pongo pygmaeus*), African green monkey (*Cercopithecus aethiops*)and squirrel monkey (*Saimiri sciureus*), respectively). In addition, the *ENVV3 *was also detected in the African green monkey by an internal ~500 bp PCR fragment (data not shown). Interestingly, sequencing of the PCR products revealed that the *ENVV2 *gene is preserved in all species analyzed and *ENVV1 *is preserved in Old World monkeys (*Cercopithecoidea*), whereas orangutan and squirrel monkey only show preservation of *ENVV*2 (Figure 2B).

We also attempted to PCR amplify the 2.5 kb fragment from the prosimian ring-tailed lemur (*Lemur catta*). However, no amplicon emerged (Figure 2A), in agreement with a database search on the accessible prosimian genome-sequencing-trace reads. In summary, both database searches and PCR amplification of *ENVV1*, *ENVV2 *and *ENVV3 *indicated an integration of ERV-V after the simian-prosimian *(Anthropoidea-Strepsirrhini*) split (77 mya[6]) and before the *Catarrhini*-*Platyrrhini *split (43 mya [6]), immediately followed by a new infection/reinfection and a duplication of the genome surrounding the ERV-V locus, or vice versa. Similarity between the 5' and 3' genomic regions surrounding the ERV-V1 and ERV-V2 loci suggests that a genomic duplication has assisted in generating ERV-V1 and ERV-V2, e.g. a 200 bp fragment upstream of HERV-V2 (chr19: 58247800–58247999, hg18) has 77% similarity to a region upstream of HERV-V1. The same is also found in chimpanzee and rhesus macaque. This similarity cannot be found in the regions flanking the ERV-V3 locus. However, the ERV-V3 locus is absent in *Hominidae*, indicating a deletion of the ERV-V3 before the human and chimpanzee separation (Figure 2B).

In theory, LTR sequence divergence can be used as a rough indicator of integration time, since the two LTRs are identical at the time of integration, but the estimate might be problematic because of confounding processes such as recombination and conversion [26,27]. The LTR divergences (human: 0.284, chimp: 0.271 and orangutan:0.370) indicate that the ERV-V locus descended from an old integration by being even more distinct than HERV-FRD (syncytin 2) LTRs (human: 0.225, chimp: 0.227 and macaque: 0.316) [28], which integrated on the same branch of the primate phylogeny. However our LTR-analysis indicates unrealistic divergence times for both ERV-V2 (96–217 mya) and syncytin 2 (83–152 mya) showing the difficulties in using LTR divergence for dating old, conserved ERV integrations.

### Gene conversion between ERV-V envelope genes

**Figure 3:**
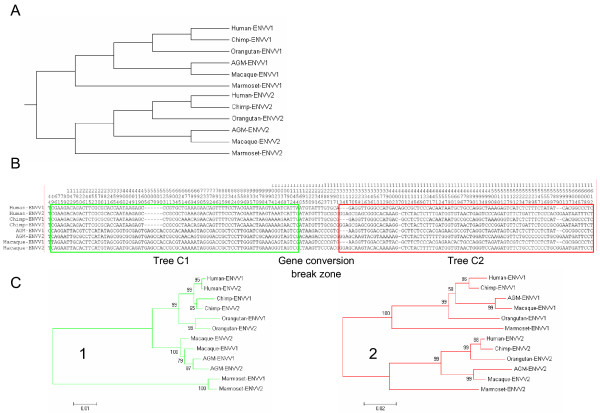
**Gene conversion between the *ENVV1 *and *ENVV2 *loci**. Detection of gene conversion between the *ENVV1 *and *ENVV2 *loci. A) Theoretical phylogenetic tree of normal species-like of evolution of *ENVV1 *and *ENVV2*. B) Alignment of sequences from species containing conserved *ENVV1 *and *ENVV2*, including variable sites only. A green box shows unexpected evolution and red box shows the expected evolution (following the phylogeny in (A)). The gene conversion break zone between the boxes is the region where 3' gene conversion breaks are predicted. C) Neighbor-joining phylogenetic tree (Maximum Composite Likelihood; 1000 Bootstrap) of the 45–1132 region (green) and the 1312-end region (red).

On the basis of the finding that both ERV-V copies can be dated back to before the *Catarrhini*-*Platyrrhini *split, we expect the evolutionary distance between the paralogue *ENVV genes *(*ENVV1-ENVV2*) to be larger than the evolutionary distance between orthologue *ENVV *genes *(ENVV1*-*ENVV1 *and *ENVV2-ENVV2*) within the most distant species (e.g. human/marmoset), resulting in two monophyletic groups, each following the primate phylogeny (an *ENVV1 *group and an *ENVV2 *group, Figure 3A). To our surprise, we observed that the paralogue genes were more closely related than the orthologue genes between the most distant species, *Hominidae*/*Cercopithecidae *and *Platyrrhini *(Kimura-2-p. distances: *ENVV1-ENVV2*, 0.024 ± 0.004 *vs ENVV2-ENVV2 *and *ENVV1-ENVV1*, 0.140 ± 0.006).

A clear change in the substitution pattern was observed in the *ENVV1/ENVV2 *alignment, and a subsequent gene-conversion test [29] yielded highly significant results (P < 0.00066, Table 1). The result indicates that gene conversion has taken place in all lineages, with 5' gene conversion breaks situated before the coding sequence or between position 1–45 as numbered from the initiator ATG, and 3' gene-conversion breaks between positions 1153–1312. To get an indication of the 5' gene conversion break, we extracted the variable sites from human, chimpanzee and macaque 5'UTRs. A shift in mutation pattern was observed around position -192, upstream of which the ERV-Vs follow the expected mutation pattern, whereas the pattern downstream of this position is consistent with gene conversion (data not shown).

**Table 1:** Test of gene conversion between ENVV1 and ENVV2

		Aligned Offsets					
ENVV1:ENVV2	P-value	Begin	End	Length	# poly	# dif	Total dif
Human	0.00000	1	1153	1153	195	2	64
Chimpanzee	0.00053	1	1312	1312	219	10	67
Orangutan	0.00003	5	1312	1308	218	19	80
African gr. monkey	0.00000	45	1296	1252	212	11	74
Macaque	0.00006	1	1290	1290	216	13	72
Marmoset	0.00066	1	1278	1278	210	12	69

P-values are Bonferroni-adjusted Karlin-Altschul p-vaules. The positions are corresponding to the aligned positions in the ENVV1 and ENVV2 sequences. "# poly" is the number of polymorphic sites in the fragment. "# dif" is the number of mismatches in the fragment. "Total dif" is the number of mismatch between *ENVV1 *and *ENVV2 *in the species.

When only looking at variable sites (Figure 3B) we note that the alignment is divided into two parts. The first part (Figure 3B, green) contains mutations that are shared by both paralogue and orthologue *ENVV*'s within the *Hominidae *and Old World monkey lineage respectively, a pattern consistent with gene conversion. The resulting phylogenetic tree (Figure 3C, tree 1) shows *ENVV *sequences clustering into monophyletic groups, where *ENVV1 *and *ENVV2 *cluster together inside each group. This is consistent with this region of the *ENVV2 *locus being transferred to the *ENVV1 *locus (or vice versa) by gene conversion. The other part (Figure 3B, red) is consistent with a model of normal species-like evolution, which is supported by the corresponding phylogenetic tree (Figure 3C, tree 2) showing paralogue *ENVV *genes splitting into two groups.

### Evidence of purifying selection of the *ENVV2 *gene

The *ENVV2 *envelope ORFs have been conserved since integration more than 40 mya (Figure 2B). In some species both ERV loci contain an intact *ENV *gene (*ENVV1 *and *ENVV2*) whereas in other species the *ENVV1 *gene is degenerated.

**Figure 4:**
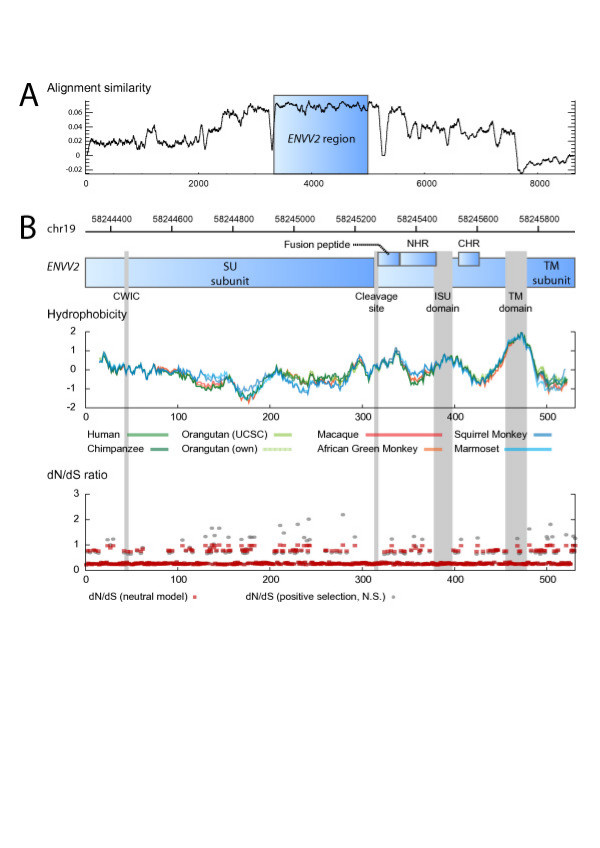
**Purifying selection of *ENVV2 *sequences**. (A) The ENVV2 reading frame (blue) is intact in all five primate genomes and shows a higher alignment similarity than the flanking regions. (B) Analysis of ENVV2 proteins, hydrophobicity plot for eight primate sequences. The most likely site specific dN/dS ratios are shown for the accepted model incorporating two classes of dN/dS ratios (red) and the non-significant model of positive selection incorporating three classes (grey).

All sequences show very high conservation within the *ENVV2 *region, whereas the similarity drops in flanking regions (Figure 4A). Additionally, the hydrophobicity profile and several characteristic motifs within gammaretroviruses are preserved in all full-length *ENVV2*s during evolution (Figure 4B). These include a CWIC motif involved in SU subunit and TM subunit interaction [30] and in controlling the fusion by a disulfide isomerization step [31], the cleavage site (RQKR) between SU and TM of the envelope gene, the hydrophobic domain in the N-terminus of TM corresponding to the fusion peptide, two heptad repeats in TM, involved in a conformational change in the envelope during fusion [32,33], the CKS-17-like immunosuppressive domain that has immunosuppressive activity [11], and the transmembrane region anchoring the envelope protein to the membrane.

**Figure 5:**
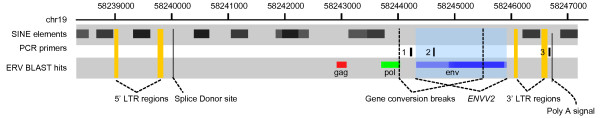
**ENVV2 annotation**. Annotation of the human HERV-V2 locus showing the high content of SINE elements in this region. *Gag*, *pol *and *env *tBLASTn hits are indicated by red, green and blue, predicted LTR regions (yellow). The region undergoing gene conversion is indicated by vertical dashed lines (see text). Splice donor site and poly A signal are predicted sites.

The HERV-V2 locus contains eight SINE elements all located closely outside *ENVV2*, both inside and between *gag *and *pol *fragments (Figure 5). Assuming a random distribution of SINE elements within the HERV-V2 locus (i.e. assuming no selection on the *ENVV2 *region), the probability of observing the 8 SINE elements outside and 0 SINE elements inside the *ENVV2 *gene is significant (*P *= 0.048), indicating that the *ENVV2 gene *has survived transposon crossfire.

The entire envelope gene shows purifying selection between all pairs of species, but not between the two almost identical orangutan sequences when testing the ratio between nonsynonymous and synonymous substitutions (dN/dS ratio, Table 2). A site-specific selection analysis using PAML [34] provides strong support for a model with two site classes (M1 vs. M0: χ^2 ^= 30.5, df = 2, *p *< 1e-7), one under strong purifying selection (mean dN/dS = 0.075, 63% of sites) and one neutral class (dN/dS = 1.000, 37% of sites). Although some positions show signs of positive selection (Figure 4B), there is no significant support for a positive selection model (M2a vs. M1: χ^2 ^= 1.46, df = 2, *p *= 0.4819).

**Table 2:** Codon-based Test of Purifying Selection between sequences

	H.s.	P.t.	P.p.a.	P.p.	M.m.	C.a.	S.s.	C.j.
H.s.		2.456	2.707	2.661	3.244	4.466	3.967	5.856
P.t.	0.008		3.852	3.750	3.665	4.862	4.290	5.939
P.p.a.	0.004	0.000		0.978	3.044	4.253	4.362	6.381
P.p.	0.004	0.000	1.000		2.986	4.143	4.167	6.225
M.m.	0.001	0.000	0.001	0.002		4.289	3.706	6.282
C.a.	0.000	0.000	0.000	0.000	0.000		4.927	7.217
S.s.	0.000	0.000	0.000	0.000	0.000	0.000		6.005
C.j.	0.000	0.000	0.000	0.000	0.000	0.000	0.000	

Test of purifying selection (codon-based Z-test). Upper triangle: Z-value and the lower triangle P-values. Analyses were conducted using the Nei-Gojobori method and Z-value variance was computed using 500 bootstrap replicates. H.s.: Human (*Homo sapiens*); P.t.: Chimpanzee (*Pan troglodytes*); P.p.a.: S. orangutan (*Pongo pygmaeus abelii*); P.p.: B. orangutan (*Pongo pygmaeus*); M.m.: Macaque (*Macaca mulatta*); C.a.: African green monkey (*Cercopithecus aethiops*); S.s.: Squirrel monkey (*Saimiri sciureus*); C.j.: Marmoset (*Callithrix jacchus*)

Additionally, *ENVV1 *and *ENVV2 *have been shown to have a placenta-specific expression in humans [18] and our RT-PCR data confirmed this particular expression (data not shown). Interestingly, EST-data from both olive baboon (*Papio anubis*, Old World monkeys) and human verify the placental expression of *ENVV1 *and *ENVV2 *[35].

## Discussion

The HERV-V1 and HERV-V2 loci display a remarkable evolution. They are the only two copies of the HERV-V family within the human genome, and there is no evidence of any solitary LTRs. However, the identified LTR regions are small and fragmented and there has been a long time span since integration. This prevents efficient recognition of solitary LTRs using BLAST, hence it is possible that they are present but remain undetected. In both HERV-V family members, the envelope genes (*ENVV1 *and *ENVV2*) consist of a long ORF. ERV-V1 and ERV-V2 are located in tandem in all species analyzed as a result of a gene duplication event. In all lineages except *Hominidae*, a third copy, ERV-V3, was detected between ERV-V1 and ERV-V2. The tandem location of this third copy is indicative of a gene triplication at the locus, however, this could not be directly proven, since no flanking sequence homologies were found.

The human *ENVV1 *shows a minor truncation at the C-terminus, due to a 1 nucleotide insert, leading to a frame shift, which has not been shown to be polymorphic in the human population, according to SNP databases (data not shown). However, the truncation has little impact on the structural prediction of the *ENVV1 *protein, nevertheless, the cytoplasmic tail is missing. Both *ENVV1 *and *ENVV2 *contain all structural motifs within envelope proteins reported to be important in viral-cell fusion. This suggests a similar function of the *ENVV *genes in the host by mediating cell-cell fusion, as has been observed for three other HERV envelopes (syncytin 1, syncytin 2 and *EnvPb1 *[15,17,18]). Phylogenetic studies show that the ERV-V family was fixed in an ancestral genome before the *Catarrhini-Platyrrhini *split ~43 mya but after simians had evolutionarily separated from the prosimians (~77 mya). Sequence analyses confirmed that *ENVV2 is *preserved among simians, and *ENVV1 *has been preserved in most species within *Catarrhini*, further all species except *Hominidae *enclose an extra ERV-V copy (ERV-V3), which seems to be lost in the *Hominidae *lineage (Figure 2B). A detailed comparative analysis of the ERV-V2 locus shows that the *ENVV2 *genes have been subjected to purifying selection (dN/dS < 1, Table 3 and Figure 4B). Additionally, *ENVV2 *survival during transposon crossfire inside the ERV-V2 locus supports purifying selection of the *ENVV2 *gene. It is unlikely that the envelope gene has been maintained as a result of retroviral replication through an extracellular cycle, since the sites of integration have been maintained through simian evolution. In conclusion, the results provide a strong argument of their beneficial role of the *ENVV2 *gene for the host.

*ENVV1 *and *ENVV2 *expression in placenta is particularly interesting since the two HERV envelope proteins (syncytin 1 and syncytin 2) having been shown to cause cell-cell fusion *in vitro *are also highly expressed in placenta [15,17]. Syncytin 1 appears to display an important role in placenta development [24] and syncytin 2 may have immunosuppressive properties in placenta [11]. Furthermore, EST data show placenta expression of *ENVV1 *and *ENVV2 *in olive baboon (Old World monkeys), which demonstrates that the placental expression is indeed conserved through simian evolution. The placenta-specific expression in combination with the purifying selection documented by our phylogenetic analysis indicates that *ENVV2 *(and/or *ENVV1*) is likely to play a physiological role in the placenta.

Comparative analysis of the two ERV-V loci suggests that gene conversion within the *ENVV *loci has taken place by DNA transfer of the major part of 5' region of *ENVV2 *to *ENVV1 *(or vice versa). Gene conversion seems to be a common event in this region, since it has taken place in all lineages (Table 1 and Figure 3). Gene conversion is a common event in evolution, and is favored between paralogue genes located on the same chromosome less than 55 kb apart [36] just as *ENVV1 *and *ENVV2*. Gene conversion has also been reported within other ERV families [27,37], however, none of them within coding viral genes. The gene conversion break points are located just upstream of the putative translation initiation site and downstream of the immunosuppressive domain (ISU) resulting in a ~1,5 kb gene conversion tract. Recent results have shown that the ISU of *ENVV2 *contains immunosuppressive activity [11], and we have shown that the ISU of *ENVV1 *and *ENVV2 *are preserved, indicating a conservation of immunosuppressive activity through evolution. Additionally, the study has shown that an envelope gene truncated after the ISU maintains the immunosuppressive activity [11]. In this manner, gene conversion in the *Hominidae *and *Cercopithecidea *lineage homogenizes the *ENVV *genes and induces two *ENVV *genes, both having a structure compatible with preservation of immunosuppressive properties.

In most simians, *ENVV1 *and *ENVV2 *have been preserved, raising the possibility of a distinct biological function of their protein products which differ in their C-terminal parts. Such function is consistent with the finding that both loci are expressed in humans as well as in olive baboon. However, this hypothesis of a separate function of the two *ENVV *genes is not supported by phylogenetic analysis in simians as such, since orangutan and New World monkeys hold only one intact *ENVV *gene.

Neither *ENVV2 *or *ENVV1 *have been shown to be fusiogenic [18], but a fusiogenic property of *ENVV *in placenta development cannot be excluded, since the selection pressure on the *ENVV2 *gene is not only restricted to the region necessary in having immunosuppressive activity, but includes the entire envelope gene. However, the fusiogenic properties may be activated by a physiological environment in placenta, like high-oxygen pressure has been found to regulate the pathway of cytotrophoblast differentiation [38,39]. Receptor recognition of the *ENVV *proteins may also be different to that of other envelope proteins such as syncytin 1, since the organization of the CWIC domain (involved in disulfide isomerisation during fusion [30,31]) (Figure 4B) within the SU is different. In *ENVV *the CWIC motif is located in the N-terminal part of the SU while it is located more C-terminally in syncytin 1 and envelope proteins of other gammaretroviruses. However this SU organization is not conflicting with envelope fusiogenic activity, since this organization is also observed in the fusiogenic syncytin 2 [10], syncytin A and syncytin B [40] ERV envelopes.

Another potential function of the *ENVV *genes could be the prevention of present-day infections by exogenous retroviruses through blocking the receptor used by the attacking virus, a process called receptor interference, as seen for other ERV envelope genes [13,14,41]. This possibility is consistent with the high sequence similarity and purifying selection of *ENVV2 *among species, but the host-receptor and the receptor binding domain of the envelope protein are unknown. Notably, the gene conversion events have preserved two copies of the presumed *ENVV *receptor binding domain, located in the N-terminal part of SU in all known retroviruses. Moreover, in all species encoding an ENVV1 protein this is predicted to be membrane-anchored and may thus potentially have receptor-interference activity. This is also the case for humans where the C-terminal truncation ENVV1 does not abrogate membrane anchoring.

## Conclusion

Our analyses have shown that the ERV-V family is small, consisting of two copies in *Hominidae*. However, orangutan, Old World monkeys and New World monkeys include an extra copy, all located in tandem on chromosome 19. The ERV-V2 envelope *ENVV2 *has been preserved for more than 43 million years in all species tested. Within *Hominidae *and Old World monkeys the *ENVV1 *has been preserved by gene conversion of a 1.5 kb region encoding the N-terminal part of ENVV2, meaning that they contain two copies of the *ENVV *envelope, both expressed in placenta. These data provide strong evidence of a beneficial role for the host in placenta. A likely function of the *ENVV *gene could be: i) Protecting the fetus against the immune system of the mother, due to immunosuppressive properties [11]. ii) Mediating cell fusion and making of the syncytiotrophoblast. iii) Blocking a retroviral receptor through receptor-interference and thereby preventing present-day retroviral infections.

## Methods

### DNA samples, PCR amplification, and sequencing

DNA from African green monkey (*Cercopithecus aethiops*), Borneo orangutan (*Pongo pygmaeus*) and squirrel monkey (*Saimiri sciureus*) was isolated from cell cultures (using Invitrogen DNAzol), tissue from ring-tailed lemur (*Lemur catta*) was a gift from Living United/Ebeltoft Zoo & Safari (DNA was isolated using a QIAGEN tissue isolation kit). All primate DNA samples were verified by amplification and direct sequence analysis of the cytochrome oxidase I (COI) gene. Sequences from human (*Homo sapiens*), chimpanzee (*Pan troglodytes*), rhesus monkey (*Macaca mulatta*), Sumatran orangutan (*Pongo pygmaeus abelii*) were extracted from the ENSEMBL genome database http://www.ensembl.org[42], marmoset sequences were extracted from the UCSC Genome Browser Primers, (1) "ENVV1/V2 forward" CAGCCTGATTTCTCACTAAACACTCCATCGAAC, "ENVV1 reverse" CTCAGCGTGCAGCGTTTCCAAGGAGGCCATCAGCG, (3) "ENVV2 reverse" CTAGTGCCTTAGTTTTTATGGGAGCT, were designed to amplify a ~2.5 kb fragment, on both sides of either *ENVV1 *or *ENVV2*. The PCR was performed by the use of expand high fidelity PCR system (Roche) in 100μl reaction using 300 nM primer, 0.2 mM dNTP, 150 ng of DNA, and buffers by the supplier. The PCR program was as follows: 1 cycle (94°C for 1 min), 30 cycles (94°C for 1 min; 60°C for 1 min; 72°C for 2.5 min), and 1 cycle (72°C for 10 min). The internal ENVV PCR was done by the same procedure by using primer (1) and the "ENVV internal" primer (2) CTAACATTTGGTTCAGGAATCC. Resulting PCR products were either directly sequenced from a 5-μl PCR mixture or after cloning of the fragment into pGEM-Teasy (Promega) using 2μl of PCR product. Sequencing were performed using the BigDye^® ^Terminator v3.1 Cycle Sequencing Kit (Applied Biosystems) (details for sequence primers are available from the authors upon request) and analyzed on an ABI3100 (Applied Biosystems). All conserved *ENVV1 *and *ENVV2 *ORFs have been deposited in GenBank under the accession numbers EU853142 and EU853155.

### LTR analysis

We tried dating the integration using the most distant species (human and marmoset) to minimize the effect of uncertainty in known speciation times. LTR regions were identified in the ERV-V2 locus in human and marmoset using dot plots, Repeatmasker data, and genomic BLAT searches. The alignment was cropped to only include sites with high alignment quality resulting in only 155–158 bp that aligned unambiguously between species. LTR divergence was calculated in MEGA4 [43] using Tamura 3- parameter distance allowing different rates among lineages (since it is easily observed that 3' LTRs are a lot more divergent than 5' LTRs between species) and different rates among sites (gamma = 1.0). The integration time is estimated as:

$${\text{Time}}_{\text{integration}}=\frac{{\text{divergence}}_{5'\text{-}3'\text{LTR}}}{{\text{rate}}_{5'\text{LTR}}+{\text{rate}}_{3'\text{LTR}}}.$$

We used species-pairs to estimate integration time from independent 5' and 3' LTR substitution rate estimates. E.g. for the human-chimp pair, we estimated the H-C 5' LTR substitution rate from the observed H-C 5' LTR divergence and published H-C speciation time (~5 mya). The human-macaque pair yields a slightly different estimate and so forth.

Sequences were aligned using ClustalW [44] and GENECONV [29] was used to predict gene conversion. We extracted variable sites using FaBox [45]. Phylogenies (Neighbor-joining phylogenetic tree, Model: Maximum Composite Likelihood; 1000 Bootstrap replicates) and evolutionary distances were calculated using MEGA4 [43].

The *ENVV2 *DNA alignment similarity plot was created using the EMBOSS plotcon program [46] with a sliding window of size 32 and EDNAFULL as similarity matrix (figure 4A). The protein hydrophobicity plots was created using EMBOSS pepwindowall program [46] with a sliding window of size 30 (figure 4B). SINE elements in the human ERV-V2 locus were identified using Repeatmasker [47] and *ENVV2 *selection tests were performed using MEGA4 [43] (Nei-Gojobori method and Z-value variance was computed using the 500 bootstrap replicates). PAML4 [34] was used to run site-specific selection tests and obtain dN/dS ratios from all *ENVV2 *sequences. The models analyzed assumed no molecular clock (clock = 0), a single omega for all tree branches (model = 0) and we used likelihood ratio tests to compare the improvement in likelihood for models incorporating 1, 2 or 3 site classes (NSsites = 0 1 2). Each analysis ran until convergence (Small_Diff = 0.5e-6) and the control file is available upon request.

## Abbreviations

ERV: Endogenous retrovirus; EST: Expressed Sequence Tags; HERV: Human endogenous retrovirus; ISU: Immunosuppressive domain; LTR: Long terminal repeats; mya: Million years ago; PCR: Polymerase chain reaction; SNP: Single-nucleotide polymorphism; SU: Surface glycoprotein; TM: Transmembrane protein

## Authors' contributions

ALK drafted the manuscript, performed all experimental work and performed the statistical and evolutionary analysis. PV participated in sequence manipulations, statistical and evolutionary analyses, prepared figures, and drafted the manuscript. LAA and FSP coordinated the study and participated in critical revision of the manuscript. All authors read and approved the final manuscript.
